# The Non-Linear Relationship between BMI and Health Care Costs and the Resulting Cost Fraction Attributable to Obesity

**DOI:** 10.3390/ijerph14090984

**Published:** 2017-08-30

**Authors:** Michael Laxy, Renée Stark, Annette Peters, Hans Hauner, Rolf Holle, Christina M. Teuner

**Affiliations:** 1 Helmholtz Zentrum München, German Research Center for Environmental Health, Institute of Health Economics and Health Care Management, 85764 Neuherberg, Germany; r.stark@helmholtz-muenchen.de (R.S.); holle@helmholtz-muenchen.de (R.H.); christina.teuner@helmholtz-muenchen.de (C.M.T.); 2 German Center for Diabetes Research, 85764 Neuherberg, Germany; peters@helmholtz-muenchen.de; 3 Helmholtz Zentrum München, German Research Center for Environmental Health, Institute of Epidemiology II, 85764 Neuherberg, Germany; 4 Technische Universität München, Klinikum Rechts der Isar, Institute for Nutritional Medicine, 81675 Munich, Germany; hans.hauner@tum.de

**Keywords:** obesity, overweight, health care costs, attributable fraction, Germany

## Abstract

This study aims to analyse the non-linear relationship between Body Mass Index (BMI) and direct health care costs, and to quantify the resulting cost fraction attributable to obesity in Germany. Five cross-sectional surveys of cohort studies in southern Germany were pooled, resulting in data of 6757 individuals (31–96 years old). Self-reported information on health care utilisation was used to estimate direct health care costs for the year 2011. The relationship between measured BMI and annual costs was analysed using generalised additive models, and the cost fraction attributable to obesity was calculated. We found a non-linear association of BMI and health care costs with a continuously increasing slope for increasing BMI without any clear threshold. Under the consideration of the non-linear BMI-cost relationship, a shift in the BMI distribution so that the BMI of each individual is lowered by one point is associated with a 2.1% reduction of mean direct costs in the population. If obesity was eliminated, and the BMI of all obese individuals were lowered to 29.9 kg/m^2^, this would reduce the mean direct costs by 4.0% in the population. Results show a non-linear relationship between BMI and health care costs, with very high costs for a few individuals with high BMI. This indicates that population-based interventions in combination with selective measures for very obese individuals might be the preferred strategy.

## 1. Introduction

With prevalence estimates of 34% for adults in the USA and 21% in Western Europe, obesity (Body Mass Index (BMI) ≥ 30 kg/m^2^) is a worldwide health problem [1]. In Germany, almost one-quarter of adult women (23.3%) and adult men (23.9%) are classified as obese [2]. The high prevalence of obesity is a concern due to various medical consequences [3] and their associated costs incurred by the increased utilisation of medical services [4].

Several studies have examined the costs of obesity by comparing the costs of persons with obesity to those without obesity, thus estimating excess costs, according to BMI groups [5]. In an earlier study, we estimated the excess costs of overweight and obese (classes I–III) compared to normal weight persons in southern Germany based on self-reported resource utilisation and measured BMI of 6731 individuals (31–96 years old) with 9070 observations [6]. Results showed that, compared to normal weight participants (18.5 kg/m^2^ ≤ BMI < 25 kg/m^2^), direct costs started to increase significantly at the obesity level II (35 kg/m^2^ ≤ BMI < 40 kg/m^2^), and indirect costs at the overweight level (25 kg/m^2^ ≤ BMI < 30 kg/m^2^).

Prior studies suggest that the relationship between BMI and health status is non-linear. In particular, the mortality risk is J- or U-shaped over BMI range, with those who are underweight (BMI < 18.5 kg/m^2^) and obese (BMI ≥ 30 kg/m^2^) facing higher mortality risk than normal weight and overweight/pre-obese individuals [7]. Evidence from the US also suggests that the association between BMI and direct health care costs is non-linear, with a steep increase in costs at higher obesity levels [8]. However, little is known about the form of this relationship in European countries.

This paper builds on our previous research and aims to analyse the potentially non-linear relationship between BMI and direct health care costs, and to quantify the cost fraction attributable to obesity in Germany.

## 2. Materials and Methods

### 2.1. Data and Study Design

The study design, data, and measures have been described in detail by Yates et al. [6]. In contrast to the analysis of Yates et al., we included underweight individuals (BMI < 18.5 kg/m^2^) and took only the last (most current) observation of participants that were examined more than once. In short, analyses are based on data of 6757 individual observations of persons aged 31–96 years from five KORA (Cooperative Health Research in the Augsburg Region) surveys from southern Germany that were conducted in 2004/2005 (F3, *n* = 3184), 2006/2008 (F4, *n* = 3080), 2008/09 (Age 1, *n* = 1079), 2010 (FoLu, *n* = 1051), and 2012 (Age 2, *n* = 822) [9]. KORA participants were drawn from the city of Augsburg and 16 adjacent communities. The study area belongs to one of the least deprived areas in Germany [10].

### 2.2. Measures

Weight and height was assessed by trained staff, and body mass index (BMI = weight [kg]/height [m]^2^) was calculated [6,9].

Direct costs due to the utilisation of health care services were estimated from a societal perspective. Resource utilisation was measured in standardised questionnaires for different time periods (3–6 months for physician visits, in- and outpatient hospital visits, and rehabilitation, and 7 days for medication) and was then extrapolated to 1 year. Annual costs were subsequently calculated by multiplying utilised resources and services with published standardised unit costs, and prices were inflated to the year 2011 [11,12,13]. Prices for the physician visits ranged from €19 for a general physician to €78 for a psychotherapist appointment. Outpatient hospital visits were priced with €40, inpatient visits with €593, and in- and outpatient rehabilitation was priced with €122 and €47, respectively. For the cost of medication, we used the pharmacy retail prices from the Scientific Institute of the *AOK* health care insurance (WIdO). Details of assigning prices for the single direct cost components have been described by Yates et al. [6].

### 2.3. Statistical Analyses

The non-linear relationship between BMI and annual health care costs were analysed using generalised additive models (GAMs).

The employed model can be notated as $Y_{i}=\beta_{0}+f_{BMI}\;\left(BMI_{i}\right)+\beta \;x_{i}^{T}+\varepsilon_{i}$, where $Y_{i}$ is the response of the individual *i*, $f_{BMI}$ is the non-parametric smooth function of the covariate BMI, $x_{i}^{T}\beta$ is the linear predictor of other covariates, and $\varepsilon_{i}$ is the error term, which is assumed to be normally distributed [14]. Due to the right skewed distribution of health care costs with many subjects having non-negative low costs and a few subjects having high costs, a gamma distribution with a log-link was specified and a hypothetical value of €1 was assigned for observations with zero costs. All regression models were adjusted for age, age^2^, gender, education level, and income, variables that were assessed in all surveys in a standardised manner. Statistical analyses were performed with the mgcv-package in the software package R (R Foundation for Statistical Computing, version 3.1.0, Vienna, Austria)).

In addition, we examined the effect on costs associated with hypothetical changes in the BMI distribution. For this, we fitted a kernel density function for BMI and then multiplied the area under the density curve for small BMI intervals (0.2 BMI units) with the estimated cost mean of each BMI interval before and after shifting/changing the BMI distribution. Concretely, we calculated the changes in health care costs in the cohort resulting from a hypothetical shift of the BMI distribution by 1, 2, and 5 BMI points to the left (BMI decrease) and by a hypothetical elimination of overweight and obesity. For the latter approach, all overweight and obese individuals were set to a BMI of 24.9 kg/m^2^ and all obese individuals were set to a BMI of 29.9 kg/m^2^.

## 3. Results

Characteristics of the analysis sample are shown in Table 1. Mean age was 59 years, 52% were women.

**Figure 1:**
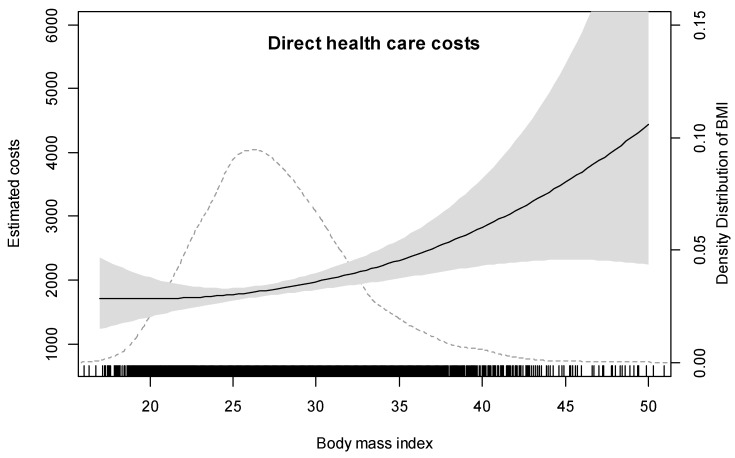
Relationship between BMI and direct health care costs. Footnote: The solid curve represents the estimated smooth functions of the non-linear association between BMI and direct health care costs using a thin plate regression spline function adjusted for age, age^2^, gender, education level, and income. The shaded areas represent approximate 95% pointwise confidence intervals. The dotted line represents the density distribution of BMI in the sample.

For total direct costs, the effective degrees of freedom (edf) for the BMI smooth term equals 2.28 and the *p*-value for the BMI smooth-term is <0.0001, indicating a significant non-linear association between BMI and direct health care costs. Figure 1 illustrates this non-linear association showing that the slope increases continuously with increasing BMI without any clear threshold. Compared to a BMI of 20, those with a BMI of 25, 30, 35, 40, 45, and 50 kg/m^2^ have 4%, 15%, 35%, 64%, 105%, and 160% higher mean health care costs, respectively. However, particularly for BMI values ≥40, confidence bands become very wide. The driving factor for this observed non-linear relationship with health care costs is the non-linear effect of BMI on hospital costs (edf = 1.88, *p*-value of BMI smooth-term <0.15) and the non-linear effect of BMI on medication costs (edf = 4.2, *p*-value of BMI smooth-term <0.01). In contrast, the association between BMI and outpatient costs is almost linear (edf = 1.01, *p*-value of BMI smooth-term <0.01). Due to the non-linear relationship, the associated reduction in annual direct health care costs for a one-unit decrease in BMI is higher for individuals with a high BMI then for individuals with a low BMI. For example, a one-point BMI reduction in an overweight person is associated with a reduction of approximately €35 in direct costs, whereas a one-point BMI reduction in a person with a BMI of 40–45 kg/m^2^ is associated with a cost reduction of approximately €135.

Shifting the BMI distribution of the whole sample by 1, 2, and 5 BMI points to the left, i.e., lowering the BMI of each individual by 1, 2, and 5 BMI points, is associated with a 2.1%, 3.9%, and 7.8% reduction in mean annual health care costs in the population. Eliminating/preventing obesity, i.e., setting all obese individuals to a BMI of 29.9 kg/m^2^, is associated with a decrease of 4.0% in annual mean health care costs, and eliminating/preventing overweight, i.e., setting all overweight and obese individuals to a BMI of 24.9 kg/m^2^, is associated with a decrease of 8.7% in annual mean health care costs in the population.

## 4. Conclusions

Detailed knowledge about the association between weight status and medical costs are needed to inform policy makers about the cost burden of excess weight and to develop cost-effective prevention strategies. This study shows that direct health care costs increase non-linearly with an increasing slope. The estimated cost fractions attributable to obesity and to overweight and obesity combined average 4% and 8.7%, respectively. These fractions are slightly higher compared to a previous study by Lehnert et al., who estimated that 3.3% of German health care expenditures for direct costs are caused by excess weight (BMI ≥ 25) in 2008 based on a top-down approach [15]. A review on cost studies in the US, however, concluded that the direct medical costs of overweight and obesity combined is 5–10% of the US health care spending [16].

Moreover, a population approach that reduces the BMI of the entire population by 2 units is associated with the same cost-reduction as eliminating/preventing obesity, i.e., a reduction of ~4% in total direct medical costs. The reason for this is that the underlying BMI distribution has very few people in the BMI range that is associated with very high costs. In light of these findings, population-based scalable behavioral or environmental interventions in combination with selective measures, such as bariatric surgery, for people with very high BMI values might be the preferred strategy [17,18].

However, these conclusions are only true if the associations reflect causal relationships and if the data is representative for the German population. In fact, the mean age of our sample is higher than that of the German population, which is about 44 years [19]. Also, our analyses were based on cross-sectional data at various time points, which is prone to confounding and only allows one to infer associations, but not causal relationships. Particularly, behavioral factors such as physical activity and environmental factors such as noise or work stress could have biased the effect estimates in either direction. More sophisticated panel design or instrumental variable approaches might be helpful to solve the endogeneity problem [8,20]. Other limitations were discussed in detail in an earlier study [6]. Most importantly, cost estimates are based on self-reported health service usage over the past 3 to 12 months and priced with average unit costs. Although this probably leads to an underestimation of resource utilisation and absolute costs [21], it is unlikely that this reporting bias depends on the weight status of participants and biased the relative effect estimates.

To our knowledge, this is the first study estimating the non-linear relationship between BMI and annual health care costs in Germany based on objectively measured height and weight data. In Germany, health insurance data does not contain BMI measurements and physician International Classification of Diseases (ICD) coding may be inconsistent and incomplete [22,23]. Thus, cost estimation based on cohort studies with standardised height and weight measurements are the method of choice to describe and analyse the economic burden of obesity and severe obesity.

## Figures and Tables

**Table 1:** Socio-demographic status of the study population.

Variable	Detail	Total
		*N* = 6757 (100%)
**Gender**	Women	3499 (51.8%)
	Men	3258 (48.2%)
**Age**	Mean	59.42 (13.8%)
**BMI**	Underweight (BMI < 18.5)	30 (0.4%)
	Normal weight (18.5 ≤ BMI < 25)	1968 (29.1%)
	Pre-obese (25 ≥ BMI < 30)	2933 (43.4%)
	Obese Class I (30 ≥ BMI < 35)	1352 (20.0%)
	Obese Class II (35 ≥ BMI < 40)	360 (5.5%)
	Obese Class III (BMI ≥ 40)	115 (1.7%)
**Education**	Basic	3831 (56.7%)
	Medium	1534 (22.7%)
	High	1392 (20.6%)
**Income**	≥150% median income	1245 (18.4%)
	≥100% and <150% median income	1913 (28.3%)
	≥60% and <100% median income	2313 (34.2%)
	<60% median income	721 (10.7%)
	Income unknown	565 (8.4%)
